# Bacterial and Fungal Keratitis in a Tertiary Care Hospital from Romania

**DOI:** 10.3390/microorganisms12040787

**Published:** 2024-04-12

**Authors:** Andrei Theodor Bălășoiu, Maria Bălășoiu, Ovidiu Mircea Zlatian, Alice Elena Ghenea

**Affiliations:** 1 Ophtalmology Department, County Clinical Emergency Hospital of Craiova, 200642 Craiova, Romania; andrei_theo@yahoo.com; 2 Ophtalmology Department, University of Medicine and Pharmacy of Craiova, 200349 Craiova, Romania; 3 Medical Laboratory, County Clinical Emergency Hospital of Craiova, 200642 Craiova, Romania; maria.balasoiu@umfcv.ro (M.B.); gaman_alice@yahoo.com (A.E.G.); 4 Microbiology Department, University of Medicine and Pharmacy of Craiova, 200349 Craiova, Romania

**Keywords:** bacterial keratitis, fungal keratitis, risk factors, etiology, antibiotic susceptibility

## Abstract

Infectious keratitis is a significant global problem that can lead to corneal blindness and visual impairments. This study aimed to investigate the etiology of infectious bacterial and fungal keratitis, identify the causative pathogens and their antimicrobial resistance patterns, and analyze the risk factors associated with the development of infectious keratitis. The study was observational and retrospective, involving 226 eyes from 223 patients presented at the Ophthalmology Clinic of the County Clinical Emergency Hospital of Craiova, Romania. The inclusion criteria included corneal ulceration/abscess/infiltrate present on slit-lamp examination and positive microbiological sampling for bacteria or fungi. The study found that the most common causes of infectious keratitis were coagulase-negative staphylococci (35.40%), *Staphylococcus aureus* (11.06%), and *Pseudomonas aeruginosa* (14.16%). The Gram-positive bacteria showed high resistance rates to penicillin, moderate rates to gentamycin and clindamycin, and low resistance to chinolones. The Gram-negative bacteria were highly resistant to ampicillin and amoxicillin–clavulanic acid, while third-generation cephalosporins, quinolones, and carbapenems were effective. Systemic antibiotics, such as vancomycine, piperacillin–tazobactam, amikacin, and ceftazidime, show promise against keratitis with low resistance rates, whereas carbapenems and topical aminoglycosides had higher resistance, leaving moxifloxacin as a potential topical option for Gram-positive bacteria and *Pseudomonas aeruginosa*, albeit with resistance concerns for *Klebsiella* spp. Although fungal keratitis was rare, *Fusarium* spp. and *Candida albicans* were the leading fungal pathogens, with incidences of 2.65% and 2.21%, respectively. *Candida albicans* was broadly susceptible to most antifungals, while *Fusarium solani*, *Curvularia lunata*, and *Alternaria alternata* exhibited resistance to many antifungals. Amphotericin B and caspofungin can be used as systemic antifungals in fungal keratitis. The study also identified risk factors for keratitis such as ocular trauma (65.92%, OR: 2.5), contact lens wear (11.94%, OR: 1.8), and corneal scarring/leukoma (10.17%, OR: 1.6). Keratitis was more frequent in individuals over 60 years old. The findings of this study have implications for the development of effective diagnostic, therapeutic, and preventive strategies for infectious keratitis.

## 1. Introduction

Eye health, including that of the cornea, is crucial for maintaining good vision and quality of life. The cornea acts as a barrier, protecting the eye from infections and providing structural support [1]. Recognizing the importance of eye health [2,3,4], the United Nations General Assembly created a resolution to prioritize vision care and eradicate preventable sight loss [5].

Infectious keratitis is a significant global problem that can lead to corneal blindness and visual impairments [6]. Understanding where infectious keratitis is most common allows for targeted public health interventions, including awareness campaigns and resource allocation, and aids in the development of new treatments and diagnostic tools.

The prevalence is higher in developing countries, particularly in regions with poor environmental and personal hygiene, low education levels, and limited access to sanitation and healthcare facilities [6,7]. Risk factors for infectious keratitis include contact lens wear, ocular trauma, ocular surface diseases (such as dry eye), facial nerve palsy, diseases of lacrimal apparatus (such as chronic dacryocystitis), post-corneal surgery (refractive procedures, penetrating or lamellar keratoplasty), corneal leukoma (traumatic or infectious), herpes simplex infections/scarring, and immunosuppressive conditions (diabetes, HIV infection, steroid treatment) [8,9,10]. Understanding the causes of infectious keratitis is necessary to improve prevention, diagnosis, and treatment strategies, and to allocate resources effectively.

Common signs of keratitis include stromal deterioration/ulcerations, which can be observed in advanced infections [11]. Diagnostic approaches for keratitis involve a combination of clinical evaluations and microbiological investigations. Microscopic examination with stains, culture, and antibiotic sensitivity testing are commonly used for diagnosis [12]. Molecular diagnostics such as polymerase chain reaction and mass spectrometry are emerging as potential tools for diagnosing keratitis [13]. Other approaches include imaging modalities such as anterior segment optical coherence tomography and in vivo confocal microscopy [14]. Genomic and metagenomic approaches [15], as well as tear proteomic analysis [16], show promise for improving the diagnosis and monitoring of infectious keratitis.

Bacteria such as *Staphylococcus aureus* and *Pseudomonas aeruginosa* have been found to adhere to and invade corneal epithelial cells, leading to infection. These bacteria can also form biofilms on contact lenses and cases, further promoting infection [12,17]. In addition, *Pseudomonas aeruginosa* has various virulence factors, such as the type III secretion system (TTSS), which allows the bacteria to inject effector proteins into host cells, leading to tissue damage [18]. The immune response plays a crucial role in limiting bacterial proliferation and protecting host tissue; however, bacteria such as *Pseudomonas aeruginosa* have mechanisms to evade and blunt the immune response, such as inhibiting the migration and function of neutrophils [18].

The early and accurate identification of bacterial pathogens in keratitis is challenging. The characteristics of bacterial and fungal keratitis are very similar, making it difficult to distinguish between them based on image analysis alone [18,19]. Additionally, the misdiagnosis of bacterial keratitis and fungal keratitis is common, with more than 30% of cases being misdiagnosed [20].

The standard diagnostic strategy in keratitis worldwide is slit lamp examination and corneal sampling/scraping, prior to any antibiotherapy (either topical or general). The current gold standard for diagnosing microbial keratitis is corneal culture; however, this method has limitations and may not always provide accurate results [21]. Polymerase chain reaction (PCR)-based molecular methods have been successful in diagnosing bacterial keratitis, but there are difficulties in obtaining bacterial cultures, particularly for mycobacteria [22]. Next-generation sequencing (NGS) has shown promise in detecting bacterial pathogens in corneal scrapings, including fastidious and dead microorganisms [23].

Regarding treatment strategy, there is no standard worldwide and there cannot be, as the etiology and resistance are different from country to country [24]. Moreover, not all antibacterial/antifungal agents can be found in all countries, which makes it even harder for a standard “strategy” to exist. The current treatment strategies for infectious keratitis have limitations, including antibiotic resistance. Antibiotic resistance is a growing concern, and broad-spectrum antibiotics are commonly used for treatment [25]. However, a combination of multiple ophthalmic antimicrobial agents may affect the efficacy of individual drugs [11,26]. Personalized treatments are needed to overcome the shortcomings of conventional formulations, which have limited ocular contact time and low therapeutic drug levels at the target ocular site [11]. Novel drug delivery strategies are being explored to improve clinical outcomes and maintain therapeutic drug levels in ocular tissues [27]. Additionally, the diverse etiologies and microbiological associations of infectious keratitis require tailored treatment approaches.

Fungal keratitis, in particular, poses considerable clinical significance due to its potential to cause severe vision impairment and blindness. It is particularly common in agricultural settings, where ocular trauma with vegetative matter is frequent. The condition can lead to severe complications, including corneal scarring, perforation, and loss of vision, underscoring its clinical significance [28]. The diagnosis of fungal keratitis is complicated by its similar presentation to other types of microbial keratitis, requiring specific laboratory tests for confirmation, indicating the need for advanced techniques such as in vivo confocal microscopy and molecular diagnostics [29].

The treatment of fungal keratitis can have various efficacies based on the fungal species involved, and resistance to antifungal agents is an emerging concern [30].

This study aimed to investigate the etiology of infectious bacterial and fungal keratitis, with a particular focus on identifying the causative pathogens and their antimicrobial resistance patterns. This study sought to understand the underlying microbial dynamics and resistance mechanisms to inform the development of more effective diagnostic, therapeutic, and preventive strategies, thereby improving clinical outcomes for patients affected by this condition. Also, the study aims to identify and analyze the risk factors associated with the development of infectious keratitis, both bacterial and fungal, including ocular trauma (65.92%), contact lens wear (11.94%), and corneal scarring/leukoma, dry eye, facial nerve palsy, chronic dacryocystitis, and corneal surgery. This involves a detailed investigation of patient demographics, underlying health conditions, environmental exposures, and clinical practices that may contribute to the incidence and severity of this ocular infection.

## 2. Material and Methods

This was an observational, retrospective study conducted on 272 eyes with a clinical diagnosis of infectious keratitis, established via slit lamp examination. The 272 eyes came from 269 adult patients (87 of whom were over 70 years old) who presented at the Ophthalmology Clinic of the County Clinical Emergency Hospital of Craiova, Romania, over a period of two years (1 January 2022–31 December 2023). None of the patients received previous eye treatments with antibiotics before presentation to the clinic. Corneal sampling was performed during slit-lamp examination prior to any topical antimicrobial or general agent administration. Samples were then sent to the Microbiology Laboratory of the Clinic of the County Clinical Emergency Hospital of Craiova, Romania, for microbiological diagnosis.

We recorded age, sex, area of residence, season of the year, fellow eye symptoms, and risk factors for keratitis. Fellow eye symptoms were the same in patients with bilateral keratitis; there were no symptoms in patients with a normal fellow eye; foreign body sensation and epiphora were the most frequent symptoms in fellow eyes (in patients with dry eye, for example). From the medical records, we collected data on the therapy applied and response to therapy. The inclusion criteria were corneal ulceration/abscess/infiltrate present on slit-lamp examination and positive microbiological sampling for bacteria or fungi. The exclusion criteria were viral or acanthamoeba keratitis (excluded via direct microscopy with Giemsa staining) or non-infectious keratitis.

To analyze the risk factors for infectious keratitis, we extracted information from the medical records of a control group of 250 patients without corneal pathology.

After microbiological sampling, the patients received empiric antimicrobial therapy with intravenous cefoperazone–sulbactam and topical tobramycin, moxifloxacin, levofloxacin, and fluconazole. The initial therapy was modified according to clinical evolution and microbiological results.

### 2.1. Microbiological Analysis

Corneal scrapings were collected on an applicator using a Bard–Parker 15 number surgical blade (Beckton-Dickinson, Mississauga, ON, Canada). The applicator was transferred onto a sterile cotton swab immersed in liquid blood heart infusion (BHI) medium (Merck KGaA, Darmstadt, Germany). BHI was sent to the laboratory and inoculated on blood agar, chocolate agar, and Sabouraud agar (Merck KGaA, Darmstadt, Germany). The media were incubated for 18 h at 37 °C in aerobic, anaerobic, and microaerophilic atmospheres obtained using the Genbag system (bioMérieux, Marcy l’Etoile, France). Gram and Giemsa smears were prepared from the developed colonies on agar culture media. Bacterial and fungal species were identified using the VITEK® 2 bacterial identification and antimicrobial susceptibility testing system (bioMérieux, Marcy l’Etoile, France), along with antibiotic susceptibility testing, using cards AST-GP67 and AST-P592 (for *Staphylococcus* spp.), AST-ST03 (for *Streptococcus viridans* group), and AST-P576 (for *Streptococcus pneumoniae*). Antibiotic susceptibility testing of Gram-negative strains was performed using the Vitek2 system with AST-N233 and AST-XN05 cards [31].

### 2.2. Antifungal Susceptibility Testing

Antifungal susceptibility was assessed using the broth microdilution technique according to the CLSI M38-A2 guidelines, 3rd edition [32]. The antifungal compounds evaluated included itraconazole (Janssen Pharmaceutica, Beerse, Belgium), voriconazole (Pfizer, Sandwich, UK), amphotericin B (Bristol Myers Squibb, Woerden, The Netherlands), isavuconazole (Basilea Pharmaceutica, Basel, Switzerland), and posaconazole (Merck KGaA, Darmstadt, Germany). Additionally, fungicidal agents such as difenoconazole, tebuconazole, and propiconazole (Sigma-Aldrich, St. Louis, MO, USA) were used. The antifungal concentrations tested ranged from 0.031 to 32 μg/mL. Fusarium strains were grown on Sabouraud glucose agar until sporulation at 30 °C, with inoculum densities adjusted to 1.8–3 × 10^6^ CFU/mL using saline with 0.05% Tween 20 for testing. The microdilution assays were incubated at 35 °C for 48 h, and the minimum inhibitory concentrations (MICs) were determined as the lowest concentrations that inhibited growth compared to the control without the drug. The reference strains Aspergillus flavus ATCC 204304, Candida parapsilosis ATCC 22019, and C. krusei ATCC 6258 were used as quality controls. MIC interpretation relied on epidemiological cutoff values (ECV). The MIC50 and MIC90 values were determined by arranging the antifungal data in ascending order and identifying the median and 90th percentile, respectively. The geometric mean MICs were calculated using Microsoft Office Excel 365 (Microsoft, Redmond, WA, USA). For MIC values outside the tested dilutions, adjustments of one log2 dilution higher or lower were made for the geometric mean calculation [32].

### 2.3. Statistical Analysis

Data were exported from the patients’ electronic records and were introduced initially in Microsoft Excel (Microsoft, Redmond, WA, USA), where the geometric means were calculated. Data were further processed using STATA 17 software (Statcorp LLc, College Station, TX, USA), where we calculated the percentages of antibiotic-resistant strains. Proportions are presented as percentages. Boxplot graphs depict the median and the first and second quartiles. Group differences were tested using exact chi square tests. The logistic regression analysis of the risk factors for keratitis was performed using a control group of patients without corneal pathology. Differences were considered significant when *p* < 0.05.

The multiple antibiotic resistance (MAR) index was calculated for each bacterial isolate to quantify its resistance profile against the tested antibiotics. The MAR index for an isolate is computed as the ratio of the number of antibiotics to which the isolate is resistant (a) to the total number of antibiotics against which the isolate was tested (b), expressed as MAR index = a/b. This index provides a numerical value reflecting the extent of resistance, with higher values indicating resistance to a larger proportion of tested antibiotics. The MAR index is crucial for identifying high-risk bacterial isolates that may act as reservoirs of resistance genes, enabling targeted interventions.

To identify potential risk factors for infectious keratitis, logistic regression analysis was employed. Each clinically relevant variable (e.g., contact lens use, history of ocular surgery, trauma, presence of ocular surface disease, etc.) was evaluated in a multivariate logistic regression model to estimate its association with the occurrence of infectious keratitis, expressed as odds ratios (ORs) with 95% confidence intervals (CIs), to adjust for potential confounders and to determine the independent effect of each risk factor on the likelihood of developing disease. The significance level for retaining variables in the model was set at *p* < 0.05.

## 3. Results

### 3.1. Etiology of Bacterial and Fungal Keratitis and Antimicrobial Resistance

Our study included 272 eyes with clinical signs of infectious keratitis and positive bacterial or fungal sampling. Of the 226 microbiologically positive samples (226/272 = 83.09%), 210 (92.92%) were from eyes with bacterial keratitis, and 16 (7.08%) were from eyes with fungal keratitis.

**Figure 1:**
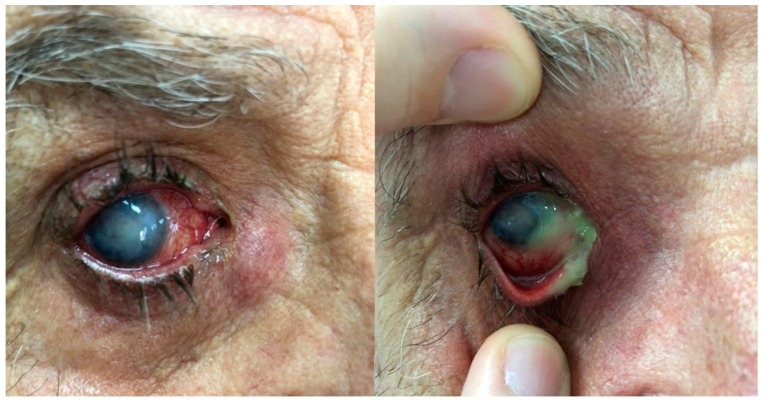
Chronic dacryocystitis keratitis with *Staphylococcus epidermidis*: clinical aspects. Intense conjunctival hyperemia, central corneal abscess, and mild inflammation at the lacrimal sac; pus on the ocular surface after lacrimal sac compression.

**Figure 2:**
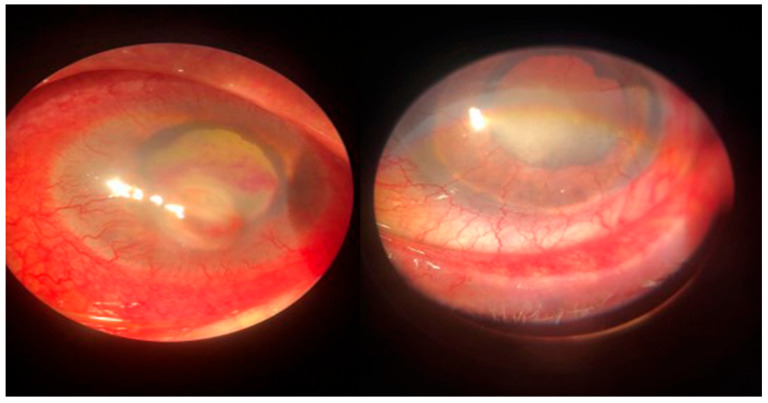
Dry eye keratitis with *Staphylococcus epidermidis*: slit-lamp aspect. Round, paracentral corneal abscess, intense conjunctival hyperemia, and neovascular panus at the periphery of the cornea; mild conjunctival hyperemia and neovascular panus in the fellow eye.

**Figure 3:**
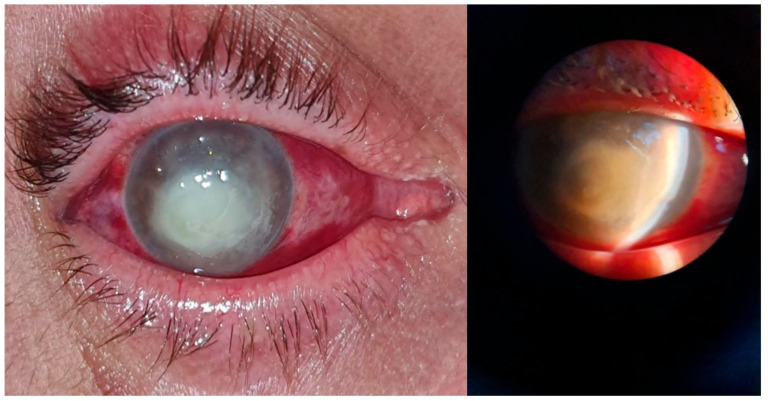
Posttraumatic keratitis with *Staphylococcus aureus*: clinical and slit-lamp aspects. Intense conjunctival hyperemia, paracentral corneal abscess, and perilesional corneal infiltration.

Coagulase-negative staphylococci (CNS) were the predominant species, representing 35.40% of the cases (Figure 1 and Figure 2), thereby underscoring its critical involvement in keratitis pathogenesis. *Staphylococcus aureus* was the next most frequent, comprising 11.06% of cases (Figure 3), further emphasizing its significant contribution to the disease.

Moreover, *Streptococcus pneumoniae* and *Streptococcus viridans* were detected in 7.08% and 3.54% of the cases, respectively, suggesting their contributory roles, albeit to a lesser degree. Among the Gram-negative bacteria, *Pseudomonas aeruginosa* was identified as a notable pathogen, accounting for 14.16% of keratitis cases, followed by *Klebsiella pneumoniae*, which was implicated in 7.96% of cases, thereby highlighting its considerable impact on the bacterial etiology of keratitis (Table 1). Additionally, other Gram-negative bacteria, including *Escherichia coli*, *Proteus mirabilis*, and various less frequently encountered organisms were also involved, although they represented a minor proportion of the cases.

**Figure 4:**
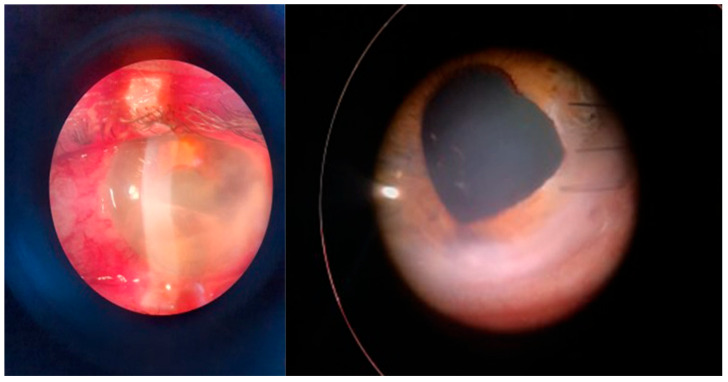
Posttraumatic keratitis with *Fusarium solani*: slit-lamp examination. Intense conjunctival hyperemia, penetrating corneal wound with corneal infiltration, and anterior chamber exudate; four weeks after surgery, the sutures are in place, quiet eye, clear central cornea, moderate scarring at the periphery.

**Figure 5:**
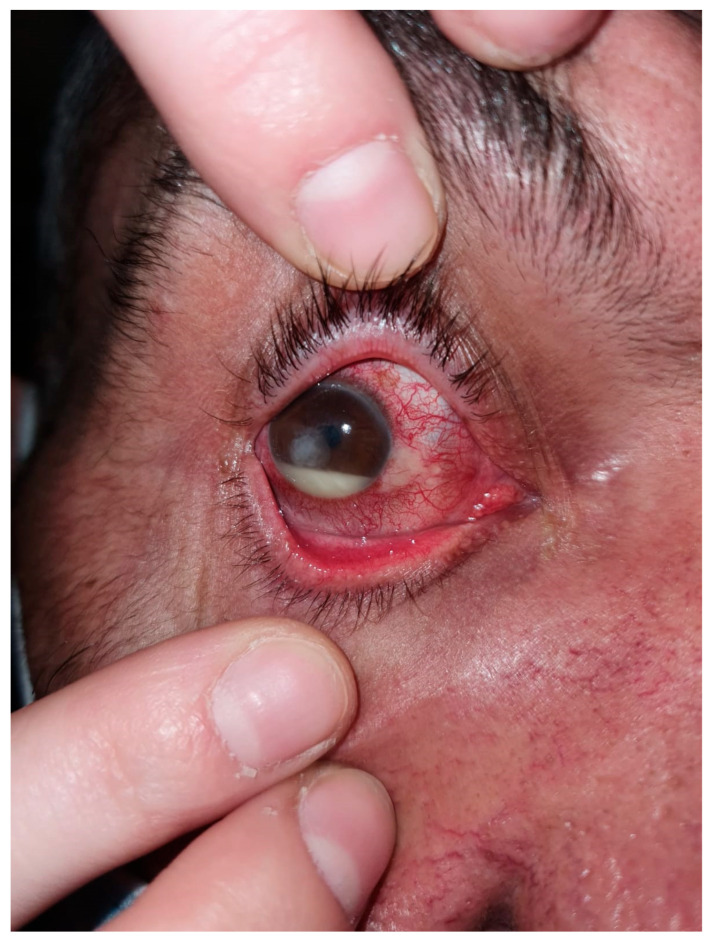
Post-scarring keratitis with *Candida albicans*: clinical aspect. Intense conjunctival hyperemia, paracentral corneal infiltration on an old corneal scar, 3 mm hypopyon in the anterior chamber.

The fungal etiology, which was less prominent than the bacterial etiology, revealed *Fusarium solani* (Figure 4) and *Candida albicans* (Figure 5) as the leading fungal pathogens, with incidences of 2.65% and 2.21%, respectively. We isolated six strains of *Fusarium solani*. The occurrence of *Aspergillus* spp., *Curvularia lunata*, and *Alternaria alternata*, although relatively rare, indicates the involvement of diverse fungi in keratitis.

### 3.2. Antimicrobial Resistance of the Isolated Microorganisms

**Figure 6:**
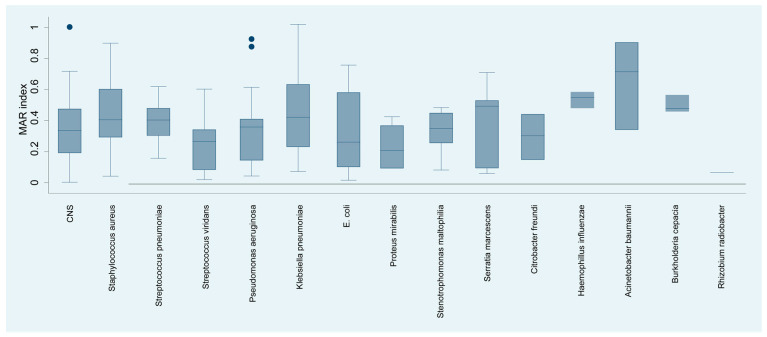
Distribution of the resistance index (MAR) by isolated bacterial species.

The analysis of the multiple antibiotic resistance (MAR) index of bacterial species isolated from keratitis cases (Figure 6) yielded a detailed profile of resistance. *Acinetobacter baumannii* exhibited the highest MAR of 65.09%, suggesting multiple antibiotic resistance, which may complicate treatment strategies. *Haemophyllus influenzae*, with an MAR index of 45.00%, showed considerable resistance.

*Staphylococcus aureus*, *Streptococcus pneumoniae*, and *Serratia marcescens* had MAR indices of 40.00%, 42.86%, and 43.00%, respectively, reflecting substantial resistance that may significantly impact the choice of empirical antibiotics. In contrast, organisms such as viridans streptococci, *Pseudomonas aeruginosa*, and *Escherichia coli* showed moderate resistance indices of 24.60%, 31.58%, and 25.53%, respectively, suggesting that while resistance is present, there may still be viable antibiotic options for treatment.

At the other end of the spectrum, *Proteus mirabilis* and *Rhizobium radiobacter* demonstrated considerably lower MAR indices of 16.35% and 6.00%, respectively, indicating a lower prevalence of resistance to the antibiotics tested.

*Staphylococcus aureus* showed substantial resistance to erythromycin (70.49%) (Table 2) and penicillin (80.65%), indicating a significant challenge in treating infections caused by this pathogen with these antibiotics. Similarly, coagulase-negative staphylococci showed a high penicillin resistance rate (77.97%).

Resistance to ciprofloxacin was observed in *Staphylococcus aureus* (33.90%) and coagulase-negative staphylococci (24.14%).

Clindamycin resistance was also notable across the pathogens tested, with resistance rates of 40.00% for *Staphylococcus aureus*, 33.33% for *Streptococcus* spp., and 33.90% for coagulase-negative staphylococci, further complicating the selection of effective antimicrobial therapy.

The resistance data also revealed a moderate resistance to gentamicin among the tested pathogens, with *Staphylococcus aureus* and coagulase-negative staphylococci showing resistance rates of 43.55% and 41.07%, respectively.

*Streptococcus pneumoniae* exhibited the following resistance rates: 81.25% to benzyl-penicillin, 68.75% to amoxicillin, 75% to sulfametoxazole/trimethoprime. Conversely, *S. pneumoniae* showed lower resistance to chloramphenicol, ceftriaxone, cefotaxime, linezolid, and vancomycin, each with a resistance rate of less than 20%. The quinolone resistance level was 31.25% to levofloxacin and 37.50% to ofloxacin, and lower resistances to moxifloxacin and sparfloxacin were observed, while resistances to erythromycin and tetracycline were higher, with resistance rates of 43.75%. The resistance to telitromycin was 18.75%, which also opens a window for the therapeutic use of this antibiotic, although its role in ocular infections is less well established.

In Gram-negative bacilli, a notable observation was the universal resistance of *E. coli*, *Klebsiella pneumoniae*, and *Citrobacter freundii* to ampicillin (Table 3), with resistance rates of 75%, 100%, and 100%, respectively. This highlights the ineffectiveness of ampicillin against Gram-negative bacteria in keratitis patients. Similarly, high resistance rates were observed for amoxicillin–clavulanic acid among *Klebsiella* spp. and *Citrobacter* spp., at 76.92% and 100%, respectively, whereas the resistance of *Pseudomonas aeruginosa* was slightly lower (66.67%), suggesting the limited utility of this beta-lactam–beta-lactamase inhibitor combination against these pathogens.

Resistance to cefoperasone-sulbactam is particularly alarming in *E. coli* and *Klebsiella pneumoniae*, with both showing a 100% resistance rate, indicating the complete failure of this first-generation cephalosporin against these bacteria in keratitis cases. Conversely, *Proteus mirabilis* showed no resistance, which could imply its potential efficacy against infections caused by this pathogen. Ceftazidime showed relatively low resistance rates across the board, with resistance ranging from 0% to 35.71%. This suggests that these antibiotics may still hold some therapeutic value against Gram-negative bacterial keratitis, although with varying efficacies across different species. Carbapenem resistance was comparably moderate across *Pseudomonas aeruginosa*, *Escherichia coli*, and *Klebsiella pneumoniae*, with rates of resistance to imipenem ranging from 20.00% to 28.57%, suggesting that carbapenems may retain utility against these pathogens.

Piperacillin-tazobactam had a 6.67% resistance for *Ps. Aeruginosa* and 14.29% resistance for *Klebsiella* spp., amikacin had 17.65% resistance for *Ps. Aeruginosa* and 21.20% resistance for *Klebsiella* spp., and third-generation cephalosporins like ceftazidimehad a resistance of 20.00% for *Ps. aeruginosa*.

Another option less suited for systemic treatment is carbapenems, as the resistance of *Klebsiella* spp. to imipenem was 28.57% and the resistance of *Ps. aeruginosa* was 22.22%.

For amikacin, while *Pseudomonas aeruginosa* showed moderate resistance at 17.65%, *Escherichia coli*, *Citrobacter* spp., and *Proteus* spp. exhibited complete susceptibility. However, *Klebsiella pneumoniae* demonstrated a resistance of 21.20%, which indicates that hospital strains have this unusual aminoglycoside resistance.

Across the spectrum, resistance to ciprofloxacin varied, with *Pseudomonas aeruginosa* at 29.41%, *Escherichia coli* at 16.67%, and *Klebsiella pneumoniae* at 23.53%. *Citrobacter freundii* and *Proteus mirabilis* exhibited 50.00% resistance and complete susceptibility, respectively. For newer-generation fluoroquinolones such as moxifloxacin and ofloxacin, complete susceptibility was observed in *Pseudomonas aeruginosa*, but varied resistance levels were noted in other species, with some instances of complete resistance in *Escherichia coli* (ofloxacin). However, *Klebsiella pneumoniae* exhibited considerably lower resistance to ofloxacin (14.29%), indicating species-specific variability in fluoroquinolone susceptibility.

The resistance data also revealed a high resistance rate to trimethoprim/sulfamethoxazole in *E. coli* (75%) and *Pseudomonas aeruginosa* (81.82%), but resistance was significantly lower in *Klebsiella pneumoniae* (33.33%), suggesting differential susceptibility patterns among Gram-negative bacteria to this antibiotic. The resistance to colistin varies significantly, with *Proteus mirabilis* showing the highest resistance at 66.67%, while *E. coli* and *Ps. aeruginosa* showed no resistance, highlighting the potential selective efficacy of colistin against specific Gram-negative pathogens in keratitis.

### 3.3. Resistance to Antifungals

The geometric means of minimal inhibitory concentrations (MICs) for various antifungal agents against a selection of fungal species implicated in infections revealed insightful trends in antifungal susceptibility (Table 4). *Fusarium solani*, the most isolated fungal species, demonstrated high MIC values for the majority of the tested agents, with amphotericin B (AMB) being an exception, showing a geometric mean MIC of 1 µg/mL, suggesting its potential effectiveness against *Fusarium solani*. In contrast, echinocandins and the azole class of antifungals, including miconazole, fluconazole, itraconazole, and voriconazole, displayed remarkably higher MICs, indicating a reduced susceptibility of *Fusarium solani* to these agents. This resistance profile is in accordance with the recognized *Fusarium* spp. profile [33].

*Candida albicans* showed considerably lower geometric mean MICs across the board, reflecting broad susceptibility to the tested antifungals. Amphotericin B, miconazole, and isavuconazole demonstrated the lowest MICs at 1 µg/mL, followed by econazole, itraconazole, posaconazole, and terbinafine, with MICs of 2 µg/mL. The echinocandin caspofungin also showed good efficacy, with an MIC of 1 µg/mL, suggesting that these antifungals are effective against *Candida albicans* infections.

*Aspergillus* spp. displayed intermediate susceptibility patterns, with Amphotericin B and caspofungin showing low MICs of 4 and 2 µg/mL, respectively. This indicates the relative effectiveness of these antifungals, while other agents, such as econazole and miconazole, showed slightly higher MICs, suggesting moderate susceptibility.

*Curvularia lunata* and *Alternaria alternata* both exhibited elevated geometric mean MICs for the majority of antifungals tested, with amphotericin B yielding MICs of 16 µg/mL and 8 µg/mL, respectively, which is higher than those observed for *Candida albicans* and *Aspergillus* spp.

The variability in the geometric mean MICs indicates a species-specific response to antifungal agents, with some fungi, such as *Candida albicans*, showing broad susceptibility, whereas others, such as *Fusarium solani*, *Curvularia lunata*, and *Alternaria alternata*, display resistance to multiple antifungal agents.

### 3.4. Analysis of Risk Factors for Bacterial and Fungal Keratitis

For analyzing the risk factors, we used a control group of 250 patients. Logistic regression analysis (Table 5) showed that among the risk factors for infectious keratitis, ocular trauma was by far the leading factor in our study, accounting for 149 eyes (65.92% of patients, OR: 2.5). The second leading risk factor in our group was contact lens wear (27 eyes, 11.94%, OR: 1.8) followed by corneal scarring/leukoma (23 eyes, 10.17%, OR: 1.6). Another important risk factor for infectious keratitis was dry eye, which accounted for 14 (6.19%, OR: 1.3) cases in the study group. The least frequent risk factors highlighted by our study were facial nerve palsy in seven eyes (3.09%, OR: 1.1), chronic dacryocystitis in four cases (1.76%, OR: 1.05), and corneal surgery (penetrating keratoplasty) in two cases (0.88%, OR: 1.02).

Three patients (1.32%) presented with bilateral keratitis, two came from contact lenses and one with previous corneal scarring. Microbiologically, all three patients with bilateral keratitis presented with bacterial etiology.

Analysis of sex and age group as risk factors. Table 6 shows a relatively balanced distribution between males and females for most pathogens, indicating that gender may not be a significant risk factor for keratitis. However, certain pathogens like *Serratia marcescens* showed a higher prevalence in females.

The data in Table 6 indicate a higher prevalence of infections in older age groups (>60 years) for most pathogens, particularly among Gram-positive bacteria. This trend might suggest an increased vulnerability to keratitis in the elderly, possibly due to age-related immune system changes or more frequent comorbidities that compromise ocular health.

Coagulase-negative staphylococci (CNS) are the most prevalent among Gram-positive bacteria, especially in the >60 years age group, suggesting that they are a significant concern in older patients. Among Gram-negative bacteria, *Pseudomonas aeruginosa* is notably prevalent, again particularly in the >60 years age group. For fungal pathogens, *Fusarium solani* and *Candida albicans* are the most common, with a slight preference for older age groups. Certain bacteria like *Haemophyllus influenzae* are exclusively found in the 18–30 years age group, suggesting that some infections might be more common in younger individuals, potentially due to lifestyle factors or different exposure risks.

Season as a risk factor. We also analyzed the etiology of infectious keratitis by season, and we observed that 55 patients presented in the cold season (October-March), and 54 patients presented in the warm season (April-September), concluding that there is no significant difference.

Patients’ occupation as a risk factor. Agriculture was the primary profession among patients with infectious keratitis (36.76%), followed by construction workers (28.92%), industry workers (20.59%), and, in lower percentages, students, intellectuals, and unemployed individuals (9.80%, 3.92%, and 9.31%, respectively) (Table 7).

### 3.5. Clinical Evolution of the Patients

Initial antibiotherapy included antibiotics from different classes (e.g., IV-administered third-generation cephalosporin, topical aminoglycoside + fluoroquinolone). Additionally, topical steroids were used in bacterial keratitis at least 24 h after initiating antibiotherapy. The results were different in eyes with bacterial keratitis than in eyes with fungal keratitis. Of the 210 eyes with bacterial etiology, 109 (51.9%) presented favorable evolution (corneal lesion decreasing in size on slit lamp examination, pain relief, increase of uncorrected visual acuity) with the initial treatment, 67 (31.9%) required treatment modification with topical netilmycin, moxifloxacin, and chloramphenicol and general imipenem/meropenem or vancomycin, and 34 (16.2%) presented unfavorable evolution regardless of treatment (corneal lesion increasing in size with perforation, phtisis bulbi, or evisceration due to pain). Fortunately, all three patients with bilateral bacterial keratitis presented with favorable outcomes with either initial therapy or modified therapy. Of the 16 eyes with fungal keratitis, 1 (6.25%) presented favorable evolution with topical and general fluconazone, 1 (6.25%) required therapy modification to general voriconazole, and 14 (87.5%) presented unfavorable evolution despite treatment.

Factors for favorable evolution (positive outcome) were young age, immunocompetent subjects, early presentation, small-sized lesion, and superficial lesion (involving anterior stroma at most). Factors for negative outcome were elderly patients, diabetes mellitus, immunosuppression, late presentation, large-sized lesion, and deep lesion (involving pre-Descemet stroma). For patients with negative outcomes, complications were decreased visual acuity (sometimes limited to hand movement or light perception), leukoma, necessity of surgery (e.g., permanent tarsorrhaphy, conjunctival flap, tectonic penetrating keratoplasty), or perforation with phthisis bulbi.

## 4. Discussion

### 4.1. Etiology of Bacterial and Fungal Keratitis and Antimicrobial Resistance

The literature shows that *Staphylococcus aureus*, *Pseudomonas aeruginosa*, *Streptococcus pneumoniae*, and *Serratia* species are key bacterial agents causing keratitis [34], which were also most frequent in our study, except for *Serratia* spp.

In this study, we observed a pronounced prevalence of Gram-positive bacteria in the etiology of bacterial and fungal keratitis. This high prevalence can be attributed to the fact that Gram-positive bacteria, such as *Staphylococcus aureus* and *Streptococcus pneumoniae*, are common inhabitants of the skin and mucous membranes, including the eye’s surface, which makes them primary candidates for opportunistic infections when the eye’s defense mechanisms are compromised. Additionally, the structure of Gram-positive thick cell walls, with their thick peptidoglycan layer, may afford them certain advantages in adhering to and penetrating the corneal surface, leading to infection. While we observed an 11.06% prevalence of *Staphylococcus aureus*, Chang et al. [35] reported a 30.7% MRSA rate and a rising resistance to fourth-generation fluoroquinolones in a 20-year study (1993–2012) in the United States. In a separate study (1996–2015), Peng et al. [36] also observed an increase in MRSA rates. Similarly, Liu et al. [37] found rising rates of antibiotic resistance among Gram-positive bacteria, including a significant increase in oxacillin resistance, in a 20 year study conducted in Taiwan.

From the Gram-negatives, the *Klebsiella* strains are lately becoming more and more frequent both in hospital and community settings, with increasing antibiotic resistance [38,39,40]. The literature reported a high-morbidity keratitis with multidrug-resistant *Pseudomonas aeruginosa* [41]. A study performed in Taiwan between 2007 and 2016 found *Pseudomonas aeruginosa* as the most frequent bacteria (35.2%), followed by *Serratia* spp. (4.6%) and *Acinetobacter* spp. (1.4%) [37]. It is worth mentioning that we isolated a strain of *Rhizobium radiobacter*, a rare cause of keratitis after corneal lesions due to vegetal matter [42,43,44].

The most frequent fungal pathogens causing infectious keratitis in our study were *Fusarium solani* (2.65%) and *Candida albicans* (2.21%). A study from France showed that yeast, particularly *Candida parapsilosis* and *C. albicans*, were the most frequently isolated fungi, accounting for 58% of the total isolates. *Aspergillus* spp. came in second, constituting 21% of the fungal isolates, while *Fusarium* sp. followed closely behind at 21% [45]. A study conducted in Shandong Province, China, found that *Fusarium* spp., particularly *F. solani*, *F. moniliforme*, and *F. oxysporum*, are the most common pathogens of fungal keratitis [46].

Other studies show that the incidence of filamentous fungi keratitis (*Aspergillus* spp., *Fusarium* spp.) varies, with the less developed countries from warm climates being the most affected. Certain regions of different continents, such as Florida, Ghana, and India, exhibit comparable climatic conditions that appear to foster the dominance of fungal keratitis [47]. In Europe, fungal keratitis is rare, with one case in Hungary and four cases in France, predominantly in agricultural workers [45].

### 4.2. Antimicrobial Resistance of the Isolated Microorganisms

Treatment-resistant bacterial keratitis poses significant challenges that emphasize the importance of studies that show the resistance pattern of bacterial species [48].

In our study, *Acinetobacter baumannii* exhibited the highest resistance (MAR = 65.09%), which is expected as it is a known hospital pathogen with multiple antibiotic resistances. The lowest resistance was identified in strains of *Proteus mirabilis* and *Rhizobium radiobacter*, potentially reflecting a higher susceptibility to standard treatment regimens. This could be explained by the fact that these were community-acquired strains. Such discrepancies in resistance levels across a spectrum of bacteria necessitate a judicious approach to antibiotic selection, emphasizing the importance of resistance profiling to inform the most effective therapeutic strategies for keratitis treatment. The variance in the MAR index emphasizes the necessity of personalized medicine and the potential need for developing alternative antimicrobial strategies or the prudent use of combination therapies to combat the rising tide of resistance in ocular infections.

In Gram-positive bacteria, the moderate level of resistance to ciprofloxacin observed in staphylococci suggests a potential limitation of the efficacy of fluoroquinolones, a commonly used class of antibiotics in ocular infections. Resistance to newer-generation fluoroquinolones, such as moxifloxacin and levofloxacin, was lower than that of older agents, such as ciprofloxacin, indicating a potential preference for these agents in treating keratitis caused by Gram-positive cocci. A study conducted in 2018 examined the prevalence of moxifloxacin resistance in *S. aureus* strains. Of the 1695 isolates tested, 33.6% were found to be resistant to moxifloxacin. Additionally, among the 621 methicillin-resistant *S. aureus* (MRSA) isolates, 72.8% exhibited resistance to moxifloxacin. For CNS, 31.1% were found to be resistant to moxifloxacin, with 51.5% of the methicillin-resistant CNS isolates displaying resistance to the drug [49]. The moderate resistance suggests that gentamicin can be a treatment option in infectious keratitis.

Concerning *Streptococcus pneumoniae* antibiotic resistance, we noted significant resistance to benzyl-penicillin, with an alarming 81.25% of the isolates exhibiting resistance to this traditional antibiotic, suggesting that the use of penicillin in its standard form may be largely ineffective against this pathogen in keratitis infections. Similarly, a high level of resistance was observed with amoxicillin and sulfametoxazole/trimethoprime, with resistance rates of 68.75% and 75.00%, respectively. The low resistance to chloramphenicol, ceftriaxone, cefotaxime, linezolid, and vancomycin, indicates that these antibiotics may still be effective treatments, albeit with the need for caution given the emerging resistance trends. Of particular interest was the moderate level of resistance to quinolones, macrolids, and tetracyclins, which might influence the selection of fluoroquinolones and macrolides in the treatment of *S. pneumoniae*-related keratitis, albeit with a moderate expectation of resistance. Furthermore, the microorganism’s resistance to newer-generation fluoroquinolones, such as moxifloxacin and sparfloxacin, was comparatively lower, suggesting the potential of these agents in the treatment of infectious keratitis.

The observed antibiotic resistance profiles among Gram-positive cocci isolated from keratitis patients underscore the importance of continuous surveillance and antibiotic stewardship in the clinical management of ocular infections.

These findings imply that the systemic use of vancomycin in infectious keratitis caused by Gram-positive cocci is recommended because the resistance is very low (0% in staphylococci and 6.25% in *Streptococcus pneumoniae*), except for streptococci from the viridans group, which had 50.00% resistance. The American Academy of Ophtalmology (AAO) also recommends vancomycin for topical use in keratitis [50]. Similarly, linezolid has very low resistance in staphylococci and moderate resistance in viridans streptococci, in which it is recommended as an alternate systemic therapy. In keratitis, third-generation cephalosporins can be efficient in systemic use because only 32.20% of staphylococci are MRSA (resistant to beta-lactam antibiotics).

Among topical treatments, fluoroquinolones can be efficient, as the resistance of *S. aureus* to levofloxacin was 20.00% and that of moxifloxacin was 16.39%, but the resistance was higher for *Streptococcus pneumoniae*. Common therapy options for keratitis include quinolones (ciprofloxacin 0.3%, levofloxacin 1.5%, and ofloxacin 0.3%), which are also recommended by the AAO [50]. Additionally, moxifloxacin 0.5% has been successfully used for a long time.

A clinical trial published in 2007 found no difference in treatment efficacy between the topical use of moxifloxacin and fortified cefazolin/tobramycin or ofloxacin [51]. Another clinical trial titled “Steroids for Corneal Ulcers Trial” (SCUT) demonstrated that the impact of moxifloxacin on microorganisms resulted in improved visual acuity after the third week of treatment [52].

Another option for topical treatment is aminoglycosides, which are not recommended because almost half of the strains are resistant.

In conclusion, the antibiotic resistance profiles among Gram-positive cocci in keratitis revealed high rates of resistance to common drugs, such as penicillin and erythromycin, moderate resistance to ciprofloxacin, and lower resistance to newer-generation fluoroquinolones and other selected antibiotics, including vancomycin.

The moderate carbapenem resistance of Gram-negatives was still higher compared with a study by Dave et al., who found imipenem as the antibiotic with the lowest resistance (17.25%) [53]. The same study found that the ceftazidime resistance was quite high (50.81%).

Other treatment options include colistin, which had a very low resistance in our study. It is worth mentioning that topical use is effective for keratitis, especially in multidrug-resistant *Ps. Aeruginosa*, while avoiding the side effects present when used systemically [48]. A study conducted by Vazirani et al. [41] revealed that the susceptibility to aminoglycosides, cephalosporins, and fluoroquinolones was less than 15% in their series of 23 patients with multidrug-resistant *Ps. aeruginosa* keratitis.

Regarding topical antibiotics, aminoglycosides such as gentamycin and tobramycin are not recommended because *Klebsiella* spp. was resistant in 54.55% of strains, and *Ps. aeruginosa* had a resistance of 58.33%. However, moxifloxacin could be an option for *Ps. aeruginosa* keratitis, although *Klebsiella* spp. had a resistance rate of 37.50%.

In conclusion, this study revealed high resistance rates of Gram-negative bacteria to traditional antibiotics such as ampicillin and ceftazidime among Gram-negative bacteria causing keratitis, emphasizing a need for the careful selection of treatment based on specific susceptibility testing.

### 4.3. Resistance to Antifungals

*Fusarium* spp., the most isolated antifungal in our study, showed the highest sensitivity to natamycin, followed by amphotericin B and terbinafine. In contrast, *Aspergillus* species, mainly *A. flavus* and *A. fumigatus*, also showed the highest sensitivity to natamycin, followed by terbinafine and amphotericin B. Both *Fusarium* spp. and *Aspergillus* spp. were relatively insensitive to ketoconazole, miconazole, itraconazole, fluconazole, and fluorocytosine [46].

Another study reported a case of fungal keratitis caused by *Aspergillus viridinutans*, which displayed distinct clinical and antifungal susceptibility patterns compared to those of *A. fumigatus*. *A. viridinutans* was resistant to amphotericin B and voriconazole, indicating the potential for varied antifungal resistance patterns even within the *Aspergillus* genus [54].

A Spanish study on the susceptibility of *Alternaria* spp. strains showed resistance to voriconazole [55], which is in accordance with our study; however, it showed high sensitivity to amphotericin B, in contrast to our results.

Our results suggest that amphotericin B may retain some efficacy against these pathogens. However, azoles and echinocandins presented high MICs, suggesting limited susceptibility of these fungal species to these classes of antifungals. An investigation into the in vitro antifungal susceptibility of 99 clinical isolates of *Curvularia species*, including *C. lunata*, against nine antifungal drugs revealed that the most active drugs were echinocandins, amphotericin B, and posaconazole, whereas voriconazole and itraconazole showed poor activity [56].

In conclusion, this study shows species-specific variations in antifungal susceptibility, with *Candida albicans* being broadly susceptible to most antifungal agents, while *Fusarium solani*, *Curvularia lunata*, and *Alternaria alternata* exhibit resistance, underscoring the need for the precise identification and susceptibility testing of fungal species to inform treatment decisions.

The differences in susceptibility to antimicrobials compared to international studies could be due to the fact that strains isolated from keratitis patients might have acquired mutations that confer resistance to some antimicrobials, which are less common in strains from other infection sites. Infections such as keratitis may promote biofilm formation on the ocular surface or within corneal tissues, and the biofilm type is more resistant than the planktonic type [57]. The unique microenvironment of the eye, including its immune response, nutrient availability, and the presence of other microbial flora, can select for more resistant pathogens. One possible explanation may be the triggering of a stress response in pathogens. This stress response can lead to the upregulation of efflux pumps as a defense mechanism to extrude toxic substances, including antimicrobial agents [57]. Patients with keratitis may have been pre-treated with topical antimicrobials, leading to selective pressure and the emergence of resistant strains. All of these resistance mechanisms promote the need for species-level identification to infer possible resistance to improve patient outcomes.

### 4.4. Analysis of Risk Factors for Bacterial and Fungal Keratitis

Many studies identify corneal trauma as a risk factor for infectious keratitis [58,59,60]. In one study, corneal trauma accounted for 91.9% of keratitis cases [59]. Another study shows that the most common causes of fungal keratitis were injuries caused by vegetal matter [59]. In our study, in contact lens wearers, *Ps. aeruginosa* predominated (51.83%), followed by *Staphylococcus aureus* (22.20%), *Serratia marcescens* (11.11%), CNS (7.41%), and *Fusarium solani* (7.41%). A study on the risk of keratitis in contact lens wearers indicates that the risk of microbial keratitis in relation to rigid gas-permeable lenses (RGPs) is approximately the same as the reference (RR∼1), while the risk is significantly lower for polymethylmethacrylate (PMMA) lenses (0.5–2.74) and daily-wear soft contact lenses (1.0–4.2). In contrast, the risk is significantly higher for extended-wear soft contact lenses (2.7–36.8) and disposable soft contact lenses (13.0–13.3). In contact lens wearers, studies indicate *Ps. aeruginosa* as the primary pathogen involved in keratitis [61]. Other ocular diseases also contributed to the risk of keratitis, with an OR greater than 30 [60].

We observed a significant correlation between the patients’ occupation and the bacterial and fungal keratitis. Agriculture workers comprised the highest percentage (36.76%), likely due to their increased exposure to environmental factors that could contribute to keratitis or different hygiene practices. Construction workers also show a substantial percentage (28.92%), possibly due to similar reasons such as exposure to dust and debris [58]. Industry workers, with a 20.59% incidence, could be exposed to specific occupational hazards depending on the nature of the industry, which might include chemicals or mechanical irritants that contribute to the development of keratitis. For example, workers in the rubber industry had a higher risk of developing keratitis due to chemical hazards, and the lesions can be then infected with microorganisms [62].

Fewer patients were students, intellectuals, and unemployed individuals (9.80%, 3.92%, and 9.31%, respectively), which might reflect reduced exposure to outdoor environmental risk factors. Also, these categories have a more frequent use of contact lens [60], which is a recognized risk factor.

### 4.5. Clinical Evolution of the Patients

Various patient-specific and pathogen-specific factors can impact clinical outcomes. The severity and the size of the corneal lesion at presentation might influence treatment outcomes, which could provide valuable insights into the prognosis. The virulence of the causative pathogens and their antimicrobial resistance profiles affect the response to therapy. Host factors such as the patient’s immune status, presence of comorbidities like diabetes, and previous history of eye diseases could play a significant role in treatment outcomes.

The clinical outcome can be influenced by adjunctive treatments such as corneal cross-linking, the use of corticosteroids, or surgical interventions like keratoplasty.

Infectious keratitis can lead to complications like corneal perforation or phthisis bulbi, including surgical interventions and their outcomes, that need to be managed according to current protocols like the preferred practice pattern of the American Academy of Ophthalmology [50].

Monitoring long-term visual outcomes and the quality of life of the patients includes monitoring the extent of visual recovery, the risk of recurrence, chronic inflammation, and other long-term complications [50].

In conclusion, this study indicates that ocular trauma is the main risk factor for infectious keratitis, followed by contact lens wear and corneal scarring, with more favorable clinical outcomes in cases of bacterial keratitis than in fungal keratitis.

Limitations. All patients came from a single hospital in the southwest region of Romania. This geographical limitation may lead to selection and recall bias, which can impact the generalizability of results. These findings might not be applicable to current or future resistance trends outside the study’s geographical context. In addition, our sample size of 226 eyes was relatively small, which can impact the results. The retrospective nature of the study implies that it relies on existing medical records and microbiological data, which may not have been collected systematically or with research purposes in mind. This could lead to inconsistencies in data quality and completeness, potentially confounding the analysis of risk factors, microbial etiologies, and antimicrobial resistance patterns.

Patients presenting at a tertiary care hospital might have more severe conditions or might have not responded to initial treatments (referral bias), which could skew the findings toward more severe cases or pathogens with higher resistance patterns. The incidence, etiology, and microbial patterns of infectious keratitis observed in this study might differ in other regions or populations, limiting the generalizability of the findings, as the patient population in this area may not be representative of broader demographics. Any limitations of the methods used for microbial identification and antimicrobial susceptibility testing could impact the accuracy of pathogen identification and antimicrobial resistance profiling. Finally, there is a difference in the local concentration depending on the method of the administration of the antimicrobial. Resistance after systemic use does not rule out efficacy after local topical application.

## 5. Conclusions

The dominance of *Staphylococcus epidermidis* and *Staphylococcus aureus* among Gram-positive bacteria and *Pseudomonas aeruginosa* among Gram-negative bacteria indicates that these organisms are primary targets for diagnostic and therapeutic strategies in bacterial keratitis. The variability in resistance patterns across different species and antibiotics emphasizes the importance of tailored antimicrobial strategies to combat keratitis effectively and ensure the judicious use of antibiotics to mitigate the risk of resistance development.

The presence of diverse fungal species, but at lower frequencies, underscores the need for fungal consideration in cases of persistent or treatment-resistant keratitis. The relatively low percentage of fungal causes compared to bacterial ones suggests that antibacterial agents are likely the primary treatment for most keratitis cases, with antifungals reserved for specific diagnoses.

Systemic antibiotics, including vancomycin, piperacillin-tazobactam, amikacin, and ceftazidime, have demonstrated effectiveness against keratitis with low resistance rates. Conversely, carbapenems and topical aminoglycosides exhibit higher resistance, making moxifloxacin a potential topical option.

The major gap in keratitis’ diagnosis and treatment is represented by negative cultures, especially after self-administrated antibiotics. In these cases, the genetic analysis of the sample may be a useful tool in detecting the infectious agent and the resistance genes. Understanding genetic mutations and expressions that confer resistance could help in developing targeted therapies that overcome resistance mechanisms.

This study emphasizes the need for standardized treatment protocols to ensure that the most effective antibiotic is selected based on the local resistance patterns, improving patient outcomes. Also, there is a need for the responsible use of antibiotics, reducing the risk of developing resistant strains and preserving the efficacy of existing antibiotics.

Research into the pathogenicity of infectious keratitis could shed light on why certain pathogens are more adept at evading host immune responses and surviving antimicrobial treatment. This could include studies on biofilm formation, immune evasion strategies, and virulence factors.

Investigating the use of combination therapies of antimicrobials with different mechanisms of action could provide a way to overcome resistance and improve treatment outcomes.

Developing advanced drug delivery systems that enhance the penetration and efficacy of antimicrobials at the site of infection could improve treatment outcomes. Research could focus on nanotechnology, targeted delivery systems, and controlled-release formulations.

The comprehensive identification of these etiological agents and the investigation of antimicrobial resistance underscores the critical necessity for the meticulous microbiological evaluation of keratitis to effectively tailor antimicrobial therapies.

## Figures and Tables

**Table 1:** Etiology of bacterial and fungal keratitis.

Species	No.	%
**Gram-positives**		
* CNS*	80	35.40%
* Staphylococcus aureus*	25	11.06%
* Streptococcus pneumoniae*	16	7.08%
* Streptococcus viridans*	8	3.54%
**Gram-negatives**		
* Pseudomonas aeruginosa*	32	14.16%
* Klebsiella pneumoniae*	18	7.96%
* Escherichia coli*	6	2.65%
* Proteus mirabilis*	6	2.65%
* Stenotrophomonas maltophilia*	4	1.77%
* Serratia marcescens*	4	1.77%
* Citrobacter freundi*	3	1.33%
* Haemophyllus influenzae*	3	1.33%
* Acinetobacter baumannii*	2	0.88%
*Burkholderia cepacia*	2	0.88%
*Rhizobium radiobacter*	1	0.44%
**Fungi**		
*Fusarium solani*	6	2.65%
*Candida albicans*	5	2.21%
*Aspergillus* spp.	2	0.88%
*Curvularia lunata*	2	0.88%
*Alternaria alternata*	1	0.44%
**Total**	**226**	**100.00%**

Coagulase-negative staphylococci (CNS) included Staphylococcus epidermidis, *S. simulans*, *S. lungdunensis*, *S. hominis*, *S. saprophyticus*, and *S. haemolyticus*. Streptococci from the viridans group included *S. mitis*, *S. mutans*, and *S. castellatus*. The *Aspergillus* species isolated were *A. flavus* and *A. tereus*.

**Table 2:** Antibiotic resistance of Gram-positive cocci strains isolated from keratitis patients.

	*Staph. Aureus* (Cards GP67, P592)	*Streptococcus* sp. (Card ST-03)	*CNS* (Cards GP67, P592)	*Streptococcus pneumoniae* (Card P576)
Amoxicillin	-	0.00%	-	68.75%
Ampicillin	-	0.00%	-	-
Chloramphenicol	-	-	-	6.25%
Ciprofloxacin	33.90%	-	24.14%	-
Clindamycin	40.00%	33.33%	33.90%	-
Ceftriaxone	-	8.53%	-	12.50%
Cefotaxime	-	8.53%	-	6.25%
Doxicycline	-	-	-	-
Erythromycin	70.49%	33.33%	49.12%	43.75%
Cefoxitin	32.20%	-	12.28%%	-
Gentamycin	43.55%	-	41.07%	-
Gentamycin HL	-	0.00%	-	-
Imipenem	-	-	-	12.50%
Linezolid	3.51%	25.00%	3.64%	6.25%
Levofloxacin	20.00%	0.00%	13.64%	31.25%
Moxifloxacin	16.39%	0.00%	11.86%	12.50%
Ofloxacin	-	-	-	37.50%
Oxacillin	32.20%	75.00%	12.28%	-
Benzyl-Penicillin	80.65%	16.67%	77.97%	81.25%
Quinupristin/Daptomycin	-	0.00%	-	-
Rifampin	9.84%	-	15.25%	-
Sparfloxacin	-	-	-	12.50%
Sulfametoxazole/Trimethoprime	-	-	-	75.00%
Telitromycin	-	-	-	18.75%
Tetracycline	66.10%	16.67%	63.79%	43.75%
Tigecylcine	43.48%	0.00%	15.00%	-
Vancomycin	0.00%	50.00%	0.00%	6.25%

CNS: coagulase-negative staphylococci.

**Table 3:** Antibiotic resistance of Gram-negative bacteria isolated from keratitis patients.

	*E. coli*	*Klebsiella pneumoniae*	*Citrobacter* spp.	*Proteus* spp.	*Pseudomonas aeruginosa*
Amikacin	0.00%	21.20%	0.00%	0.00%	17.65%
Amoxicillin-clavulanic acid	33.33%	76.92%	100.00%	25.00%	66.67%
Ampicillin	75.00%	100.00%	100.00%	50.00%	-
Aztreonam	40.00%	33.33%	33.33%	0.00%	22.22%
Cefuroxime	25.00%	53.85%	0.00%	0.00%	55.56%
Cefalotine	-	-	-	-	-
Cefixime	-	-	-	-	-
Cefpirome	25.00%	50.00%	50.00%	0.00%	40.00%
Ceftazidime	20.00%	35.71%	0.00%	25.00%	20.00%
Ceftazidime-avibactam	-	-	-	-	-
Ceftolozane-tazobactam	-	-	-	-	-
Cefotaxime	0.00%	30.77%	50.00%	0.00%	-
Gentamycin	25.00%	54.55%	0.00%	16.67%	58.33%
Tobramycin	25.00%	53.85%	50.00%	33.33%	27.78%
Ciprofloxacin	16.67%	23.53%	50.00%	0.00%	29.41%
Levofloxacin	0.00%	0.00%	0.00%	0.00%	-
Moxifloxacin	50.00%	37.50%	0.00%	0.00%	0.00%
Ofloxacin	100.00%	14.29%	50.00%	0.00%	0.00%
Imipenem	20.00%	28.57%	33.33%	50.00%	22.22%
Ertapenem	0.00%	31.25%	0.00%	0.00%	41.67%
Meropenem	0.00%	50.00%	0.00%	0.00%	16.67%
Piperacillin					
Piperacillin-tazobactam	33.33%	14.29%	0.00%	0.00%	6.67%
Sulfametoxazole-trimethoprim	75.00%	33.33%	0.00%	0.00%	81.82%
Colistin	0.00%	7.69%	33.33%	66.67%	0.00%
Tetracycline	100.00%	100.00%	100.00%	50.00%	-
Chloramphenicol	0.00%	25.00%	50.00%	33.33%	-
Tigecycline	0.00%	41.67%	66.67%	25.00%	25.00%

Antibiotic susceptibility testing of Gram-negative bacteria was performed using the Vitek2 system with AST-N233 and AST-XN05 cards.

**Table 4:** Geometric means of the minimal inhibitory concentration (µg/mL) of antifungal agents.

Species	AMB	ECO	MCZ	FLU	ITR	FCY	NYS	CAS	VOR	POS	ISA	TEB
*Fusarium solani*	1	64	32	64	64	64	64	16	64	64	64	64
*Candida* *albicans*	1	2	1	4	2	4	32	1	4	2	1	2
*Aspergillus* spp.	4	8	4	2	4	8	64	2	8	4	2	2
*Curvularia lunata*	16	32	64	32	32	64	64	16	64	64	64	64
*Alternaria* *alternata*	8	16	32	32	64	64	64	8	64	64	64	64

AMB: amphotericin B; ECO: econazole; MCZ: miconazole; FLU: fluconazole; ITR: itraconazole; FCY: flucitozine; NYS: nystatin; CAS: caspofungin; VOR: voriconazole; POS: posaconazole; ISA: isaconazole; TEB: tebuconazole.

**Table 5:** Multivariate logistic regression analysis of risk factors for bacterial and fungal keratitis.

Risk Factor	Odds Ratio (OR)	95% CI	*p*-Value
Ocular Trauma	2.5	1.8–3.4	<0.001
Contact Lens Wear	1.8	1.2–2.7	0.004
Corneal Scarring/Leukoma	1.6	1.0–2.5	0.038
Dry Eye	1.3	0.7–2.4	0.4
Facial Nerve Palsy	1.1	0.5–2.3	0.8
Chronic Dacryocystitis	1.05	0.3–3.6	0.95
Corneal Surgery (PK)	1.02	0.2–5.1	0.98

**Table 6:** Etiology of bacterial and fungal keratitis by sex and age group of the patients.

	Age Group No.	(% Total)					
Species	18–30 Years	30–45 Years	46–60 Years	>60 Years	Males	Females	Total
**Gram-positives**							
* * *CNS*	4 (5.19%)	5 (6.49%)	2 (2.60%)	66 (85.71%)	37 (48.05%)	40 (51.95%)	77 (100%)
* * *Staphylococcus aureus*	6 (24.00%)	1 (4.00%)	2 (8.00%)	16 (64.00%)	12 (48.00%)	13 (52.00%)	25 (100%)
* * *Streptococcus pneumoniae*	1 (6.25%)	2 (12.50%)	1 (6.25%)	12 (75.00%)	9 (56.25%)	7 (43.75%)	16 (100%)
* * *Streptococcus viridans*	1 (12.50%)	1 (12.50%)	1 (12.50%)	5 (62.50%)	5 (62.50%)	3 (37.50%)	8 (100%)
**Gram-negatives**							
* * *Pseudomonas aeruginosa*	9 (28.12%)	4 (12.50%)	0 (0.00%)	19 (59.38%)	17 (53.12%)	15 (46.88%)	32 (100%)
* * *Klebsiella pneumoniae*	5 (27.78%)	3 (16.67%)	4 (22.22%)	6 (33.33%)	7 (38.89%)	11 (61.11%)	18 (100%)
* * *Escherichia coli*	0 (0.00%)	0 (0.00%)	5 (83.33%)	1 (16.67%)	3 (50.00%)	3 (50.00%)	6 (100%)
* * *Proteus mirabilis*	0 (0.00%)	0 (0.00%)	0 (0.00%)	6 (100%)	3 (50.00%)	3 (50.00%)	6 (100%)
* * *Stenotrophomonas maltophilia*	0 (0.00%)	0 (0.00%)	1 (25.00%)	3 (75.00%)	1 (25.00%)	3 (75.00%)	4 (100%)
* * *Serratia marcescens*	0 (0.00%)	0 (0.00%)	1 (25.00%)	3 (75.00%)	0 (0.00%)	4 (100%)	4 (100%)
* * *Citrobacter freundi*	0 (0.00%)	3 (100.00%)	0 (0.00%)	0 (0.00%)	2 (66.67%)	1 (33.33%)	3 (100%)
* * *Haemophyllus influenzae*	3 (100%)	0 (0.00%)	0 (0.00%)	0 (0.00%)	2 (66.67%)	1 (33.33%)	3 (100%)
* * *Acinetobacter baumannii*	0 (0.00%)	0 (0.00%)	0 (0.00%)	2 (100%)	2 (50.00%)	2 (50.00%)	2 (100%)
*Burkholderia cepacia*	0 (0.00%)	0 (0.00%)	1 (50.00%)	1 (50.00%)	1 (50.00%)	1 (50.00%)	2 (100%)
*Rhizobium radiobacter*	0 (0.00%)	1 (100%)	0 (0.00%)	0 (0.00%)	0 (0.00%)	1 (100%)	1 (100%)
**Fungi**							
*Fusarium solani*	1 (16.67%)	2 (33.33%)	0 (0.00%)	3 (50.00%)	3 (50.00%)	3 (50.00%)	6 (100%)
*Candida albicans*	0 (0.00%)	0 (0.00%)	1 (20.00%)	4 (80.00%)	1 (20.00%)	4 (80.00%)	5 (100%)
*Aspergillus* spp.	0 (0.00%)	0 (0.00%)	1 (50.00%)	1 (50.00%)	2 (100%)	0 (0.00%)	2 (100%)
*Curvularia lunata*	0 (0.00%)	2 (100%)	0 (0.00%)	0 (100%)	1 (50.00%)	1 (50.00%)	2 (100%)
*Alternaria alternata*	1 (100%)	0 (100%)	0 (0.00%)	0 (100%)	0 (0.00%)	1 (100%)	1 (100%)
**Total**	**31 (13.90%)**	**24 (10.76%)**	**20 (8.96%)**	**148 (66.36%)**	**108 (48.43%)**	**115 (51.57%)**	**223 (100%)**

Coagulase-negative staphylococci (CNS) included Staphylococcus epidermidis, *S. simulans*, *S. lungdunensis*, *S. hominis*, *S. saprophyticus*, and *S. haemolyticus*. Streptococci from the viridans group included *S. mitis*, *S. mutans*, and *S. castellatus*. The *Aspergillus* species isolated were *A. flavus* and *A. ereus*. We also isolated six strains of *Fusarium solani*.

**Table 7:** Etiology of bacterial and fungal keratitis by occupation of the patients.

	Agriculture Workers	Construction Workers	Students	Industry Workers	Intellectuals	Unemployed	
No.	75	59	20	42	8	19	223
%	36.76%	28.92%	9.80%	20.59%	3.92%	9.31%	100%

Coagulase-negative staphylococci (CNS) included Staphylococcus epidermidis, *S. simulans*, *S. lungdunensis*, *S. hominis*, *S. saprophyticus*, and *S. haemolyticus*. Streptococci from the viridans group included *S. mitis*, *S. mutans*, and *S. castellatus*. The *Aspergillus* species isolated were *A. flavus* and *A. tereus*. We also isolated six strains of *Fusarium solani*.

## Data Availability

Data are contained within the article.

## References

[1] Dolan P., Laffan K., Kudrna L. (2021). The Welleye: A Conceptual Framework for Understanding and Promoting Wellbeing. Front. Psychol..

[2] Ravilla T., Karumanchi S., Das T. (2021). Health Management and Information: Key Principles and Enablers in Eye Health Program. South-East Asia Eye Health.

[3] Burton M.J., Ramke J., Marques A.P., Bourne R.R.A., Congdon N., Jones I., Ah Tong B.A.M., Arunga S., Bachani D., Bascaran C. (2021). The Lancet Global Health Commission on Global Eye Health: Vision beyond 2020. Lancet Glob. Health.

[4] Assi L., Rosman L., Chamseddine F., Ibrahim P., Sabbagh H., Congdon N., Evans J., Ramke J., Kuper H., Burton M.J. (2020). Eye Health and Quality of Life: An Umbrella Review Protocol. BMJ Open.

[5] Martines E., Reitberger H., Chow C., Brun P., Zuin M., Fuchsluger T.A. (2018). Perspectives in Ophthalmology. Comprehensive Clinical Plasma Medicine: Cold Physical Plasma for Medical Application.

[6] Sereda D., Nieścior H., Metelska A., Metelski J., Szwed M. (2022). Risk Factors for Infectious Keratitis—A Literature Review. J. Educ. Health Sport.

[7] Stapleton F. (2023). The Epidemiology of Infectious Keratitis. Ocul. Surf..

[8] Ting D.S.J., Ho C.S., Deshmukh R., Said D.G., Dua H.S. (2021). Infectious Keratitis: An Update on Epidemiology, Causative Microorganisms, Risk Factors, and Antimicrobial Resistance. Eye.

[9] Ung L., Chodosh J. (2023). Urgent Unmet Needs in the Care of Bacterial Keratitis: An Evidence-Based Synthesis. Ocul. Surf..

[10] Pramanick P., Sengupta M., Banerjee M., Ghosh S., Mitra A.N., Chakraborty M., Sengupta M. (2022). Microbiological Profile in Patients Having Keratitis in a Tertiary Care Hospital in India. Cureus.

[11] Dunster E., Johnson W.L., Wozniak R.A.F. (2023). Antimicrobial Drug-Drug Interactions in the Treatment of Infectious Keratitis. Cornea.

[12] Suzuki T., Inoue H. (2022). Mechanisms Underlying Contact Lens-Related Keratitis Caused by Pseudomonas Aeruginosa. Eye Contact Lens.

[13] Badenoch P.R. (2019). A Turning Point for Contact Lens-Associated Microbial Keratitis?. Clin. Exp. Ophthalmol..

[14] Bakken I.M., Jackson C.J., Utheim T.P., Villani E., Hamrah P., Kheirkhah A., Nielsen E., Hau S., Lagali N.S. (2022). The Use of in Vivo Confocal Microscopy in Fungal Keratitis—Progress and Challenges. Ocul. Surf..

[15] Borroni D., Bonzano C., Sánchez-González J.M., Rachwani-Anil R., Zamorano-Martín F., Pereza-Nieves J., Traverso C.E., García Lorente M., Rodríguez-Calvo-de-Mora M., Esposito A. (2023). Shotgun Metagenomic Sequencing in Culture Negative Microbial Keratitis. Eur. J. Ophthalmol..

[16] Winiarczyk M., Biela K., Michalak K., Winiarczyk D., Mackiewicz J. (2022). Changes in Tear Proteomic Profile in Ocular Diseases. Int. J. Environ. Res. Public. Health.

[17] Eby A., Hazlett L., Microb J., Technol B. (2015). Pseudomonas Keratitis, a Review of Where Wehave Been and What Lies. J. Microb. Biochem. Technol..

[18] Gao N., Kumar A., Yu F.S.X. (2015). Matrix Metalloproteinase-13 as a Target for Suppressing Corneal Ulceration Caused by Pseudomonas Aeruginosa Infection. J. Infect. Dis..

[19] Wu J., Yuan Z., Fang Z., Huang Z., Xu Y., Xie W., Wu F., Yao Y.F. (2023). A Knowledge-Enhanced Transform-Based Multimodal Classifier for Microbial Keratitis Identification. Sci. Rep..

[20] Mayya V., Shevgoor S.K., Kulkarni U., Hazarika M., Barua P.D., Acharya U.R. (2021). Multi-Scale Convolutional Neural Network for Accurate Corneal Segmentation in Early Detection of Fungal Keratitis. J. Fungi.

[21] Tuft S., Bunce C., De S., Thomas J. (2023). Utility of Investigation for Suspected Microbial Keratitis: A Diagnostic Accuracy Study. Eye.

[22] Üçkayabaşi A., Kandemİr T., Nağiyev T. (2022). Investigation of the Presence of Mycobacteria along with Microbial Agents in Cases of Keratitis. Turk. Bull. Hyg. Exp. Biol..

[23] Badreldin I., Justesen B., Lyhne N., Fuursted K., Vestergaard A.H., Justesen U.S. (2023). Identification of Microorganisms in Patients with Keratitis by Next-Generation Sequencing. Acta Ophthalmol..

[24] Herrmann W., Kohnen T., Erfurth U., Kohnen T. (2018). Bacterial Keratitis. Encyclopedia of Ophthalmology.

[25] Shilovskikh O.V., Ponomarev V.O., Kazaykin V.N., Tkachenko K.A. (2023). Bacterial Keratitis. Part 2. Topical Aspects of Treatment. Oftalmologiya.

[26] Sarkar T., Singh M., Kumar Jana B., Mazumder B. (2023). Current Formulation Strategies to Design Novel Carriers for Targeted Drug Delivery and Management of Infectious Keratitis: A Comprehensive Review on the Present State of the Art. Lett. Drug Des. Discov..

[27] Onkar A. (2023). Commentary: Tackling Childhood Infectious Keratitis. Indian J. Ophthalmol..

[28] Mahmoudi S., Masoomi A., Ahmadikia K., Tabatabaei S.A., Soleimani M., Rezaie S., Ghahvechian H., Banafsheafshan A. (2018). Fungal Keratitis: An Overview of Clinical and Laboratory Aspects. Mycoses.

[29] Ansari Z., Miller D., Galor A. (2013). Current Thoughts in Fungal Keratitis: Diagnosis and Treatment. Curr. Fungal Infect. Rep..

[30] Holz F.G., Tadayoni R., Beatty S., Berger A., Cereda M.G., Hykin P., Staurenghi G., Wittrup-Jensen K., Altemark A., Nilsson J. (2016). Key Drivers of Visual Acuity Gains in Neovascular Age-Related Macular Degeneration in Real Life: Findings from the AURA Study. Br. J. Ophthalmol..

[31] Jin W.Y., Jang S.J., Lee M.J., Park G., Kim M.J., Kook J.K., Kim D.M., Moon D.S., Park Y.J. (2011). Evaluation of VITEK 2, MicroScan, and Phoenix for Identification of Clinical Isolates and Reference Strains. Diagn. Microbiol. Infect. Dis..

[32] Clinical & Laboratory Standards Institute (2017). CLSI M38: Reference Method for Broth Dilution Antifungal Susceptibility Testing of Filamentous Fungi.

[33] Alastruey-Izquierdo A., Cuenca-Estrella M., Monzón A., Mellado E., Rodríguez-Tudela J.L. (2008). Antifungal Susceptibility Profile of Clinical *Fusarium* spp. Isolates Identified by Molecular Methods. J. Antimicrob. Chemother..

[34] Lakhundi S., Siddiqui R., Khan N.A. (2017). Pathogenesis of Microbial Keratitis. Microb. Pathog..

[35] Chang V.S., Dhaliwal D.K., Raju L., Kowalski R.P. (2015). Antibiotic Resistance in the Treatment of Staphylococcus Aureus Keratitis: A 20-Year Review. Cornea.

[36] Peng M.Y., Cevallos V., McLeod S.D., Lietman T.M., Rose-Nussbaumer J. (2018). Bacterial Keratitis: Isolated Organisms and Antibiotic Resistance Patterns in San Francisco. Cornea.

[37] Liu H.Y., Chu H.S., Wang I.J., Chen W.L., Hu F.R. (2019). Microbial Keratitis in Taiwan: A 20-Year Update. Am. J. Ophthalmol..

[38] Ghenea A.E., Cioboată R., Drocaş A.I., Țieranu E.N., Vasile C.M., Moroşanu A., Țieranu C.G., Salan A.I., Popescu M., Turculeanu A. (2021). Prevalence and Antimicrobial Resistance of Klebsiella Strains Isolated from a County Hospital in Romania. Antibiotics.

[39] Ghenea A.E., Zlatian O.M., Cristea O.M., Ungureanu A., Mititelu R.R., Balasoiu A.T., Vasile C.M., Salan A.-I., Iliuta D., Popescu M. (2022). TEM,CTX-M,SHV Genes in ESBL-Producing Escherichia Coli and Klebsiella Pneumoniae Isolated from Clinical Samples in a County Clinical Emergency Hospital Romania-Predominance of CTX-M-15. Antibiotics.

[40] Bucataru A., Balasoiu M., Ghenea A.E., Zlatian O.M., Vulcanescu D.D., Horhat F.G., Bagiu I.C., Sorop V.B., Sorop M.I., Oprisoni A. (2023). Factors Contributing to Surgical Site Infections: A Comprehensive Systematic Review of Etiology and Risk Factors. Clin. Pract..

[41] Vazirani J., Wurity S., Ali M.H. (2015). Multidrug-Resistant Pseudomonas Aeruginosa Keratitis: Risk Factors, Clinical Characteristics, and Outcomes. Ophthalmology.

[42] Barker N.H., Thompson J.M., Mullen M.G., Weekes M.A., Nguyen L.N., Haynes C.K.M., Miller D. (2016). Rhizobium Radiobacter: A Recently Recognized Cause of Bacterial Keratitis. Cornea.

[43] Balasoiu A.T., Zlatian O.M., Ghenea A.E., Davidescu L., Lungu A., Golli A.L., Udriștoiu A.L., Balasoiu M. (2022). A Rare Case of Endophthalmitis with Rhizobium Radiobacter, Soon after a Resolved Keratitis: Case Report. Antibiotics.

[44] Fenner B.J., Kumar A., Tan N.Y.Q., Ang M. (2019). Case of Isolated Rhizobium Radiobacter Contact Lens-Related Infectious Keratitis: A Plant Microbe Now Emerging as a Human Pathogen. Am. J. Ophthalmol. Case Rep..

[45] Rondeau N., Bourcier T., Chaumeil C., Borderie V., Touzeau O., Scat Y., Thomas F., Baudouin C., Nordmann J.-P., Laroche L. (2002). Fungal Keratitis at the Centre Hospitalier National d’Ophtalmologie Des Quinze-Vingts: Retrospective Study of 19 Cases. J. Fr. Ophtalmol..

[46] Xie L., Zhai H., Zhao J., Sun S., Shi W., Dong X. (2008). Antifungal Susceptibility for Common Pathogens of Fungal Keratitis in Shandong Province, China. Am. J. Ophthalmol..

[47] Dóczi I., Gyetvai T., Kredics L., Nagy E. (2004). Involvement of *Fusarium* spp. in Fungal Keratitis. Clin. Microbiol. Infect..

[48] Egrilmez S., Yildirim-Theveny Ş. (2020). Treatment-Resistant Bacterial Keratitis: Challenges and Solutions. Clin. Ophthalmol..

[49] Thomas R.K., Melton R., Asbell P.A. (2019). Antibiotic Resistance among Ocular Pathogens: Current Trends from the ARMOR Surveillance Study (2009–2016). Clin. Optom..

[50] Lin A., Rhee M.K., Akpek E.K., Amescua G., Farid M., Garcia-Ferrer F.J., Varu D.M., Musch D.C., Dunn S.P., Mah F.S. (2019). Bacterial Keratitis Preferred Practice Pattern^®^. Ophthalmology.

[51] Constantinou M., Daniell M., Snibson G.R., Vu H.T., Taylor H.R. (2007). Clinical Efficacy of Moxifloxacin in the Treatment of Bacterial Keratitis. A Randomized Clinical Trial. Ophthalmology.

[52] Oldenburg C.E., Lalitha P., Srinivasan M., Manikandan P., Jayahar Bharathi M., Rajaraman R., Ravindran M., Mascarenhas J., Nardone N., Ray K.J. (2013). Moxifloxacin Susceptibility Mediates the Relationship between Causative Organism and Clinical Outcome in Bacterial Keratitis. Investig. Ophthalmol. Vis. Sci..

[53] Dave T.V., Dave V.P., Sharma S., Karolia R., Joseph J., Pathengay A., Pappuru R.R., Das T. (2019). Infectious Endophthalmitis Leading to Evisceration: Spectrum of Bacterial and Fungal Pathogens and Antibacterial Susceptibility Profile. J. Ophthalmic Inflamm. Infect..

[54] Shigeyasu C., Yamada M., Nakamura N., Mizuno Y., Sato T., Yaguchi T. (2012). Keratomycosis Caused by Aspergillus Viridinutans: An Aspergillus Fumigatus-Resembling Mold Presenting Distinct Clinical and Antifungal Susceptibility Patterns. Med. Mycol..

[55] Alastruey-izquierdo A., Cuesta I., Ros L., Mellado E., Rodriguez-tudela J.L. (2011). Antifungal Susceptibility Profile of Clinical *Alternaria* spp. Identified by Molecular Methods. J. Antimicrob. Chemother..

[56] da Cunha K.C., Sutton D.A., Fothergill A.W., Gené J., Cano J., Madrid H., de Hoog S., Crous P.W., Guarro J. (2013). In Vitro Antifungal Susceptibility and Molecular Identity of 99 Clinical Isolates of the Opportunistic Fungal Genus Curvularia. Diagn. Microbiol. Infect. Dis..

[57] Cannon R.D., Lamping E., Holmes A.R., Niimi K., Baret P.V., Keniya M.V., Tanabe K., Niimi M., Goffeau A., Monk B.C. (2009). Efflux-Mediated Antifungal Drug Resistance. Clin. Microbiol. Rev..

[58] Bourcier T., Thomas F., Borderie V., Chaumeil C., Laroche L. (2003). Bacterial Keratitis: Predisposing Factors, Clinical and Microbiological Review of 300 Cases. Br. J. Ophthalmol..

[59] Bharathi M.J., Ramakrishnan R., Meenakshi R., Shivakumar C., Raj D.L. (2009). Analysis of the Risk Factors Predisposing to Fungal, Bacterial & Acanthamoeba Keratitis in South India. Indian J. Med. Res..

[60] Liesegang T.J. (1997). Contact Lens-Related Microbial Keratitis: Part I: Epidemiology. Cornea.

[61] Kolar S.S.N., Luca V., Baidouri H., Mannino G., McDermott A.M., Mangoni M.L. (2015). Esculentin-1a(1-21)NH2: A Frog Skin-Derived Peptide for Microbial Keratitis. Cell. Mol. Life Sci..

[62] Groves J.S., Smail J.M. (1969). Outbreak of Superficial Keratitis in Rubber Workers. Br. J. Ophthalmol..

